# Asafœtida No Preventive of Measles

**Published:** 1846-06

**Authors:** 


### FACTS FROM CORRESPONDENTS.

---

### ASAFŒTIDA NO PREVENTIVE OF MEASLES.

Dr. Stahl, of Quincy, Illinois, thinks it worth his while to show, that Dr. Harper (see vol. 3, this Journal, new series, p. 455) has not made out the claims of asafœtida to be regarded as a preventive of measles. We make the following extracts from his letter, deeming any lengthened discussion of the subject quite unnecessary.

"In order to prove anything a preventive, we must first prove that measles is contagious, which I think would be very difficult to do, and secondly, we must prove that when it rages epidemically, every person who has not had the disease, must necessarily be attacked by it. To examine these two points, however, would lead me beyond the limits which I set for this note. My object is merely to disprove the arguments of Dr. Harper by my own direct and but recent experience. Neither myself nor any of my children ever had the measles. During the recent prevalence of that disease in this city and neighborhood, I attended and saw at least one hundred cases in all its various stages, handled the patients while they were in perspiration, sat on the side of their beds; in short, was in the closest contact with them, and not seldom in ten minutes after thus handling a patient with measles, and without washing my hands or changing my clothes, I would nurse or play with my own children, and *yet neither myself nor any of my children have as yet manifested the least symptoms of the disease.*

"Had I or my family, during the prevalence of the disease, used any soi-disant preventive, I might, like Dr. Harper, have been induced to give it the credit of our protection."

April 10th, 1846.

